# Differentiating low-risk thymomas from high-risk thymomas: preoperative radiomics nomogram based on contrast enhanced CT to minimize unnecessary invasive thoracotomy

**DOI:** 10.1186/s12880-024-01367-5

**Published:** 2024-08-01

**Authors:** Chao Gao, Liping Yang, Yuchao Xu, Tianzuo Wang, Hongchao Ding, Xing Gao, Lin Li

**Affiliations:** 1 Department of Medical Imaging, The Fourth Affiliated Hospital of Harbin Medical University, Harbin, China; 2 Department of PET-CT, Harbin Medical University Cancer Hospital, Harbin, China; 3 School of Nuclear Science and Technology, University of South China, Hunan Harbin, China; 4 Department of Medical Imaging, Heilongjiang Red Cross Hospital, Harbin, China; 5 Department of Physical Diagnosis, Heilongjiang Provincial Hospital, Harbin, China

**Keywords:** Thymic tumors, Quantitative radiomics analysis, Computed tomography (CT), WHO staging system

## Abstract

**Background:**

This study was designed to develop a combined radiomics nomogram to preoperatively predict the risk categorization of thymomas based on contrast-enhanced computed tomography (CE-CT) images.

**Materials:**

The clinical and CT data of 178 patients with thymoma (100 patients with low-risk thymomas and 78 patients with high-risk thymomas) collected in our hospital from March 2018 to July 2023 were retrospectively analyzed. The patients were randomly divided into a training set (*n* = 125) and a validation set (*n* = 53) in a 7:3 ratio. Qualitative radiological features were recorded, including (a) tumor diameter, (b) location, (c) shape, (d) capsule integrity, (e) calcification, (f) necrosis, (g) fatty infiltration, (h) lymphadenopathy, and (i) enhanced CT value. Radiomics features were extracted from each CE-CT volume of interest (VOI), and the least absolute shrinkage and selection operator (LASSO) algorithm was performed to select the optimal discriminative ones. A combined radiomics nomogram was further established based on the clinical factors and radiomics scores. The differentiating efficacy was determined using receiver operating characteristic (ROC) analysis.

**Results:**

Only one clinical factor (incomplete capsule) and seven radiomics features were found to be independent predictors and were used to establish the radiomics nomogram. In differentiating low-risk thymomas (types A, AB, and B1) from high-risk ones (types B2 and B3), the nomogram demonstrated better diagnostic efficacy than any single model, with the respective area under the curve (AUC), accuracy, sensitivity, and specificity of 0.974, 0.921, 0.962 and 0.900 in the training cohort, 0.960, 0.892, 0923 and 0.897 in the validation cohort, respectively. The calibration curve showed good agreement between the prediction probability and actual clinical findings.

**Conclusions:**

The nomogram incorporating clinical factors and radiomics features provides additional value in differentiating the risk categorization of thymomas, which could potentially be useful in clinical practice for planning personalized treatment strategies.

**Supplementary Information:**

The online version contains supplementary material available at 10.1186/s12880-024-01367-5.

## Introduction

Thymomas are relatively rare neoplasms but are the most frequent indolent tumors of the anterior mediastinum, accounting for up to 50% of anterior mediastinal masses [1–6]. The World Health Organization (WHO) classification is extensively used in formulating clinical treatment strategies and evaluating prognosis for thymoma patients. It divides thymomas into five subtypes: A, AB, B1, B2, and B3 [7, 8]. The pathological subtype of thymomas is closely related to distinct personalized therapeutic schedules [9–11]. Thoracotomy is the main curative treatment for subtypes B2 or B3, while subtypes A, AB, or B1 can be cured by less-invasive bronchoscopy thymectomy [12, 13]. Additionally, patients with subtypes B2 or B3 often suffer from an increased risk of various complications, resulting in a lower 5-year survival rate and more frequent clinical recurrence rate [14, 15]. Therefore, the WHO classification of thymomas was further simplified into low-risk thymomas (LRT) consisting of types A, AB, and B1, and high-risk thymomas (HRT) composed of types B2 and B3 to meet actual clinical requirements [7, 9, 16, 17].

Currently, the non-invasive imaging technique available for assessing thymomas is mainly contrast-enhanced computed tomography (CE-CT) due to its relatively high convenience, spatial resolution, and ability to provide information on tumor dynamic blood supply [2, 5]. Although some researchers have demonstrated that CE-CT could provide valuable information for evaluating thymomas, the radiological performance of different subtypes largely overlaps, which could lead to misdiagnosis and inappropriate treatment strategies [18].

Radiomics, with the high-throughput extraction and integration of quantitative signatures from standard-of-care medical images, provides significant clinical usefulness and enables non-invasive assessment of tumor heterogeneity [19, 20]. Currently, radiomics analysis has been widely applied in early diagnosis, disease screening, and prognosis evaluation, assisting clinicians in making individualized treatment strategy decisions for patients [21, 22]. However, to our best knowledge, there are few studies focusing on the prediction of risk categorization of thymomas based on radiomics analysis. Therefore, we aimed to develop a CE-CT-based radiomics nomogram for the preoperative prediction of risk grades in patients with thymomas.

## Materials and methods

### Patients’ recruitment

**Fig. 1:**
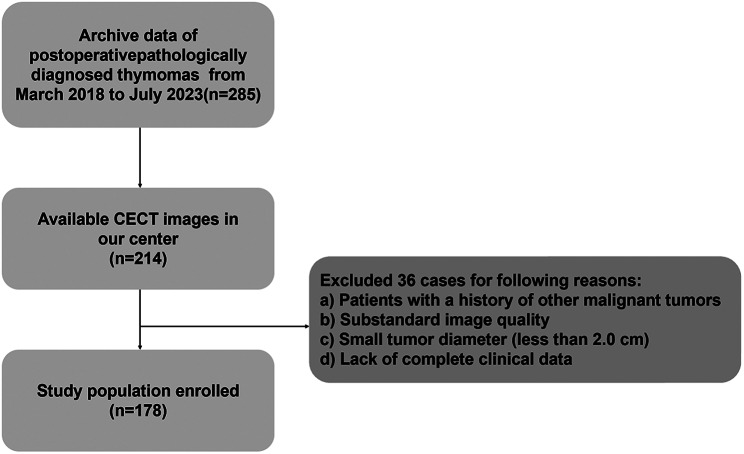
Patients selection flow diagram

The retrospective research protocol was reviewed, approved, and overseen by the institutional review board of the Fourth Affiliated Hospital of Harbin Medical University, and the need for written informed consent was waived. Patients who underwent surgical resection with pathological confirmation of thymomas between March 2018 and July 2023 were retrospectively reviewed. The inclusion criteria were as follows: (1) histological diagnosis confirmed by surgical resection; (2) available preoperative contrast-enhanced computed tomography (CE-CT) images (within 1 month prior to surgery); (3) the longest diameter of the lesion greater than 2.0 cm; and (4) no relevant treatment (chemotherapy, radiation therapy, or surgery) performed before CE-CT scans. The exclusion criteria were: (1) patients with a history of other malignant tumors; (2) absence of a preoperative CE-CT scan; and (3) substandard image quality, such as motion artifacts. Finally, a total of 178 patients were included in the study, and 75 patients were excluded (Fig. 1).

### Image acquisition

All chest CT images were obtained from Discovery CT 750 HD (GE Medical Systems, USA). Detailed scanning parameter were as follows: tube voltage 100-140 kV and tube current 350-550 mA, slice thickness 3 mm, reconstruction interval 3 mm, matrix size 512 × 512 and field of view 450 mm. The non-ionic contrast media (Iohexeol, 350 mg/ml, GE, Boston, USA) had been administered at a rate of 3.0-3.5 ml/s and 1.2 ml/kg to the patients. The arterial phase (AP) and venous phase (VP) images were scanned around 30s and 60s post injection of contrast media, respectively.

### Image analysis

One experienced radiologist who has 10-year practicing experience, blinded to the surgical and pathological results, independently evaluated the CECT images for thymoma size, location, shape, capsule, calcification, necrosis, fatty infiltration, lymphadenopathy and enhanced CT value. Final results were re-confirmed by a senior radiologist who has 15-year specialized experience in the chest disease diagnosis and any discrepancy was settled through consensus by discussion.

### Image segmentation and feature extraction

Three steps were adopted to preprocess the CT images before feature extraction, which was executed following a method detailed in our earlier publications [23, 24]. Firstly, all CT images were resampled to a uniform voxel size of 1 mm × 1 mm × 1 mm using linear interpolation to minimize the influence of different layer thicknesses. Secondly, the continuous images were converted into discrete values based on the gray-scale discretization process (bin width = 25). Finally, the Laplacian of Gaussian and wavelet image filters were used to eliminate the mixed noise in the image digitization process to obtain low- or high-frequency features. Axial CT Digital Imaging and Communications in Medicine images were applied for tumor segmentation.

**Fig. 2:**
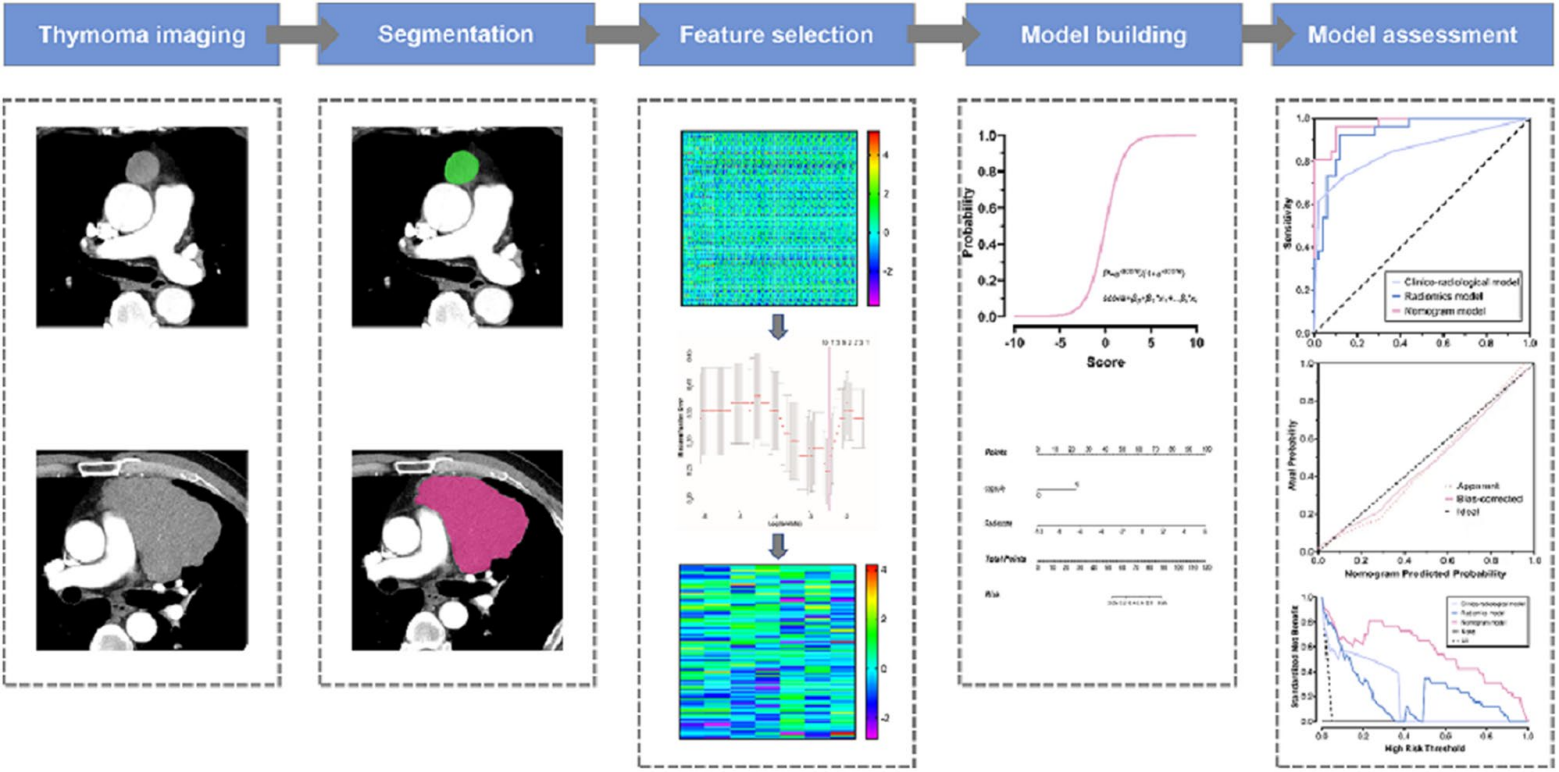
The radiomics analysis workflow

The tumor lesion was delineated on axial CT images using ITK-SNAP software (version 3.6.0, www.itksnap.org). Each region of interest (ROI) was manually contoured along the boundary of tumors on the maximum cross-sectional imaging under circumstances that all the adjacent tissues had been carefully avoided. To evaluate the intra- and inter-observer reproducibility, intra- and inter-class correlation coefficients (ICCs) were calculated. Two readers drew the ROIs on 40 randomly selected CT images (20 cases of LRT and 20 cases of HRT). Reader 1 repeated the segmentations two weeks later. An ICC greater than 0.80 indicated good agreement with feature extraction. Therefore, only radiomics features with ICC > 0.80 were selected for further radiomics analysis. The volume of interest (VOI) segmentation for the remaining cases was performed by reader 1. The process of tumor segmentation and feature extraction was presented in Fig. 2.

**Fig. 3:**
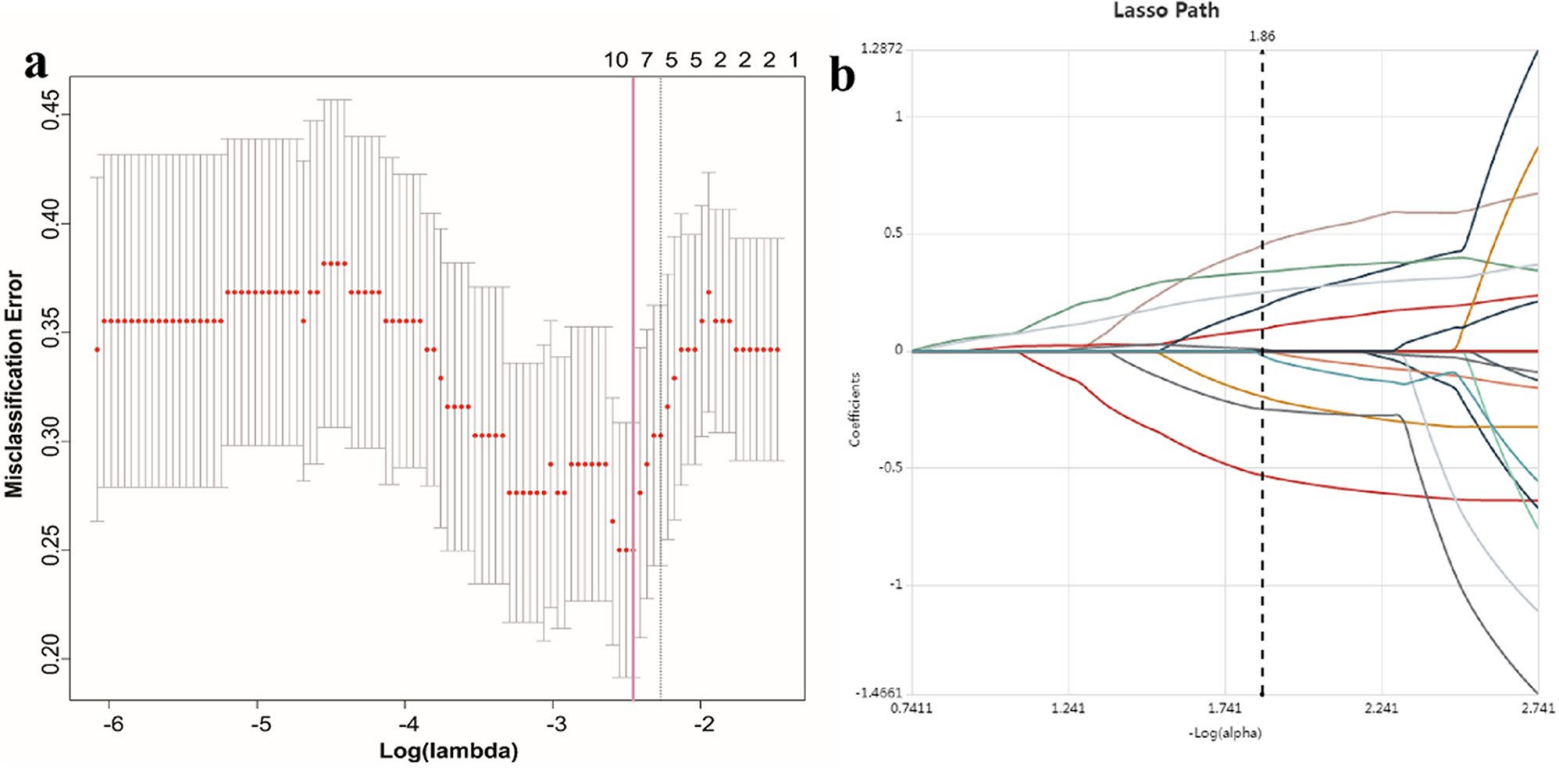
Selection of significant parameters in radiomics features in the training cohort and definition of linear predictor. (**a**) Ten time cross-validation for tuning parameter selection in the LASSO model. (**b**) The LASSO coefficient profiles of the seven non-zero-coefficient features

Radiomics features were extracted from each CT-derived VOI by applying dedicated AK software (Artificial Intelligence Kit, GE Healthcare). A sum of 851 radiomics features were extracted from VOIs in the AP and VP images of each patient, respectively. The extracted radiomics features included (i) first-order feature, (ii) shape-based feature, (iii) gray level cooccurrence matrix, (iv) gray level run length matrix, (v) gray level size zone matrix, (vi) neighboring gray tone difference matrix and (vii) gray level dependence matrix [25]. The details of radiomics features were listed in supplementary data. The process of radiomics features selection was displayed in Fig. 3.

### Radiomics signature selection

Radiomics feature selection was done in 851 features extracted from each VOI of the CT image. To improve predictive performance of the radiomics model and avoid overfitting, dimension reduction was performed based on reproducibility and redundancy. Firstly, only the radiomics features with ICC value ≥ 0.80 were chosen for further analysis. Secondly, univariate logistic regression analysis was utilized to select features with *P*-value < 0.05 for the subsequent analysis. Thirdly, multivariate logistic regression analysis was utilized to choose features closely related to thymoma risk categorization. Finally, the most discriminative features were retained using the least absolute shrinkage and selection operator (LASSO) method. LASSO model was applied to improve the diagnostic accuracy and interpretability of the prediction model by altering the model fitting process to choose only a subset of extracted features for final model construction. LASSO regression shrinks the coefficient estimates toward zero, with the degree of shrinkage dependent on an additional parameter, alpha. To determine the optimal values for alpha, a 10-time cross-validation was used, and we chose alpha via the minimum criteria and a value of ln (alpha)= -2.4 was chosen.

### Model’s development and diagnostic performance assessment

For differentiating HRT from LRT, independent clinical factors and radiological features based on the univariable and multivariable logistic regression analysis were used to develop clinico-radiological model. A radiomics model was also constructed and a radiomics score (Rad-score) was calculated. A combined radiomics nomogram, integrating clinical–radiological parameters and Rad-score was built. Diagnostic efficacy of the different models was assessed by accuracy, sensitivity, specificity and area under curve (AUC). The calibration curve was applied to assess the agreement between the prediction results of the nomogram and the actual clinical findings, and decision curve analysis (DCA) was performed to estimate the clinical usefulness of the radiomics nomogram.

### Statistics

Statistical analysis was performed using R-studio and GraphPad Prism software. The Kolmogorov-Smirnov test was applied to evaluate whether the data conforms to the normal distribution or not, if so, then the continuous variables were summarized with means ± standard deviations. Consecutive and categorical variables were tested by Student’s T test (or Mann-Whitney U test) and Chi-square test (or Fishers’exact test), respectively. The receiver operating characteristic (ROC) curve was constructed to assess the discriminative performance of each model. A two-sided *P* value < 0.001 was considered as statistically significant difference.

## Results

### Clinical characteristics

A total of 178 patients with thymomas were enrolled, composed of 84 males and 94 females (ranged from 29 to 75 years old). According to the histological and immunohistochemical results, among the pathological subtypes of the World Health Organization, there were 100 patients with LRT (type A: 28; type AB: 40; type B1: 32) and 78 patients with HRT (type B2: 36; type B3: 42). There were no significant differences in sex, age, location, tumor diameter, calcification, lymphadenopathy, and enhanced CT value between patients in the training cohort and the validation cohort, as indicated in Table 1 (all *P* values > 0.001). Nevertheless, for patients with LRT and HRT, significant statistical differences were observed in shape, capsule integrity, necrosis and fatty infiltration (all *P* values < 0.001).

**Table 1:** Baseline clinical characteristics of patients

	Training cohort (*n* = 125)			Validation cohort (*n* = 53)		
	**LRT (n = 72) HRT(n = 53)**		*P*value	**LRT (n = 28) HRT(n = 25)**		*P*value
**Sex(n(%))**			0.302			0.801
Male	32(38.0)	21(38.0)		16(38.0)	15(38.0)	
Female	40(72.0)	32(38.0)		12(38.0)	10(38.0)	
**Age (years)**	54 ± 9	47 ± 10	0.559	54 ± 11	50 ± 8	0.704
**Diameter (mm)**	40.9 ± 15.2	42.3 ± 14.8	0.605	44.7 ± 13.6	47.1 ± 12.9	0.586
**Shape(n(%))**						
Round/Oval	46	19	<0.001	20	10	0.004
Irregular	26	34		8	15	
**Location(n(%))**			0.522			0.496
Middle	34	28		3	10	
Left/Right	39	25		38	18	
**Capsule integrity(n(%))**			<0.001			<0.001
Complete	54	15		21	5	
Incomplete	18	38		7	20	
**Calcification(n(%))**						0.289
Present	17	14	0.101	8	8	
Absent	55	39		20	17	
**Necrosis(n(%))** Present	23	38	<0.001	8	6	<0.001
Absent	55	15		20	19	
**Fatty infiltration(n(%))** Present	0	15	<0.001	0	9	<0.001
Absent	72	38		28	16	
**Lymphadenopathy(n(%))** Present	4	6	0.157	3	7	0.102
Absent	68	47		25	18	
**Enhanced CT value(HU)**	75.2 ± 13.1	78.5 ± 18.2	0.069	70.9 ± 17.4	72.3 ± 15.6	0.078

*Note* continuous variables are expressed as Median (interquartile range). Otherwise, data are number of patients. HU (Hounsfieldunit)

### Clinico-radiological model

Regarding clinical variables and conventional radiological features, after univariate and multivariate logistic regression analysis, only incomplete capsule represented independent predictive variable of thymoma risk stratification. Then, the clinico-radiological model was constructed based on the above independent variables. The results of the univariate analysis and multivariate logistic regression analysis are displayed in Table 2. The AUC, accuracy, sensitivity, and specificity of the clinico-radiological model based on the above independent factor was 0.851 (95% CI, 0.830–0.871), 0.855, 0.615, and 0.980 for the training cohort and 0.801(95% CI, 0.754–0.829), 0.765, 0.607, and 0.945 for the validation cohort (Table 3).

**Table 2:** Univariate and multivariate analysis to identify independent predictors for thymoma risk stratification

	Univariate logistic regression			Multivariate logistic regression		
	OR	95% CI	*P* value	OR	95% CI	*P* value
Sex	0.91	[0.72–1.35]	0.443	NA	NA	NA
Age	1.23	[0.75-1.94]	0.305	NA	NA	NA
Diameter	1.96	[0.49-5.02]	0.600	NA	NA	NA
Shape	0.92	[0.90-0.95]	<0.001	0.97	[0.94-1.04]	0.389
Location	1.04	[0.40–2.11]	0.512	NA	NA	NA
Capsule integrity	1.04	[1.03–1.06]	<0.001	1.04	[1.01–1.07]	<0.001
Calcification	0.48	[0.20–1.17]	0.105	NA	NA	NA
Necrosis	1.21	[1.07–1.38]	<0.001	1.08	[0.88-1.40]	0.402
Fatty infiltration	1.10	[1.04–1.18]	<0.001	1.12	[0.96-1.26]	0.093
Lymphadenopathy	0.55	[0.21–1.35]	0.153	NA	NA	NA
Enhanced CT value	2.12	[0.92–4.85]	0.071	NA	NA	NA

**Table 3:** Predictive performance of training and validation cohorts for different models

Models	Training cohort				Validation cohort			
	AUC(95%CI)	ACC	SEN	SPE	AUC(95%CI)	ACC	SEN	SPE
Clinico-radiological model	0.851 (0.830–0.871)	0.855	0.615	0.980	0.801 (0.754–0.829)	0.765	0.607	0.945
Radiomics model	0.932 (0.912–0.949)	0.895	0.923	0.880	0.923 (0.871–0.937)	0.882	0.884	0.870
Nomogram model	0.974 (0.965–0.989)	0.921	0.962	0.900	0.960 (0.950–0.975)	0.892	0.923	0.871

*Note AUC*, Area under the curve; *CI*, Confidence interval; *ACC*, Accuracy; *SEN*, Sensitivity; *SPE*, Specificity

### **Feature extraction**,** selection**,** and prediction performance** of radiomics model

A total of 851 radiomics features were initially extracted from each VOI of the CT image. The intra-observer ICC ranged from 0.805 to 0.955, and inter-observer ICCs ranged from 0.740 to 0.912. Subsequently, a sum of 748 radiomics features were selected when using ICC ≥ 0.80 as the repeatability standard. Univariate and multivariate logistic regression analyses were further performed to reduce the dimensions of these features. Then, seven features with non-zero coefficients were selected for inclusion in the score calculation formula using LASSO logistic regression. Finally, the radiomics signature was developed, including a radiomics score calculation formula as follows:

Rad-score= -1.8832-0.8610*original_glcm_MaximumProbability (AP)-0.361*wavelet.HHH_glcm_MCC(AP)-2.1960*wavelet.LLH_ngtdmComplexity(AP)-1.1123*wavelet.LLH_gldm_DependenceNonUniformitNormalized(AP) + 1.0852*wavelet.HHL_firstorder_Median (VP) + 0.3025*original_shape_Flatness(VP) + 1.1029*wavelet.LHH_firstorder_Skewness (VP).

The radiomics prediction model was built based on seven significant radiomics features. This model exhibited AUC of 0.932 (95% CI 0.912–0.949) in the training cohort with sensitivity, specificity, and accuracy of 0.923, 0.880, and 0.895 respectively. Applied in the validation cohort, the model yielded AUC of 0.923 (95% CI 0.871–0.937) with sensitivity, specificity, and accuracy of 0.884, 0.870, and 0.882, respectively (Table 3).

### Development and validation of the nomogram

**Fig. 4:**
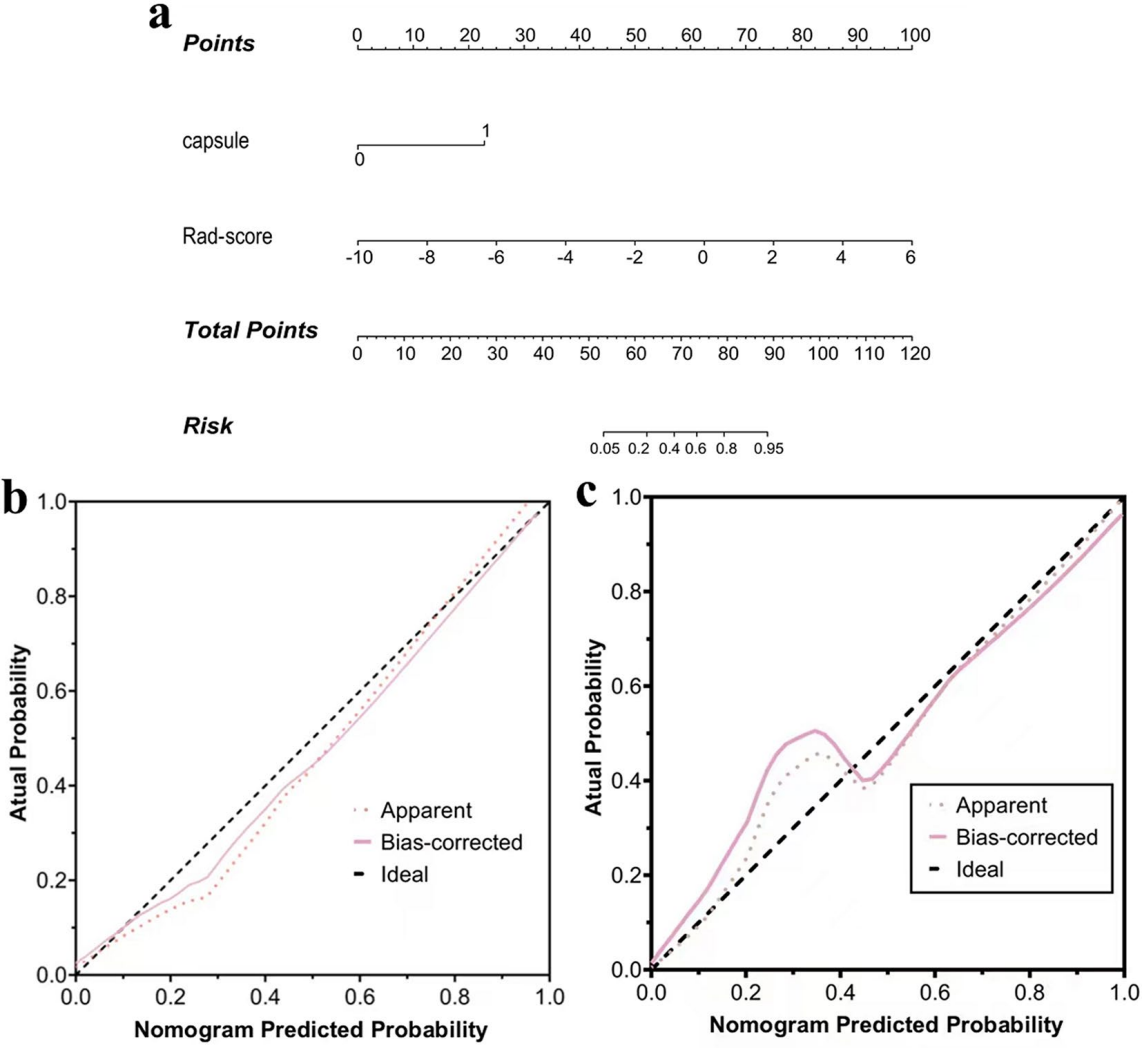
Combined radiomics nomogram for the prediction of thymoma risk stratification. (**a**). Radiomics nomogram was developed in the training cohort for the prediction of thymoma risk stratification, with capsule integrity and radiomics signature incorporated. (**b, c**). The calibration curves analysis of clinico-radiological model, radiomics model and radiomics nomogram (b training cohort, *n* = 125; c validation cohort, *n* = 93). The 45° line represents a perfect match between the actual (Y-axis) and the probability of differential diagnosis, clinico-radiological model, radiomics model and radiomics nomogram (X-axis)

To develop a more precise and clinically applicable tool to predict thymomas risk stratification, we applied the logistic regression algorithm to build a combined radiomics nomogram incorporating capsule integrity and CE-CT radiomic features as presented in Fig. 4a. This nomogram model exhibited an area under the curve (AUC) of 0.974 (95% CI 0.965–0.989) in the training cohort, with a sensitivity of 0.962, specificity of 0.900, and accuracy of 0.921. When applied to the validation cohort, the nomogram model yielded an AUC of 0.960 (95% CI 0.950–0.975), with a sensitivity of 0.923, specificity of 0.871, and accuracy of 0.892 (Table 3). The AUC values of the training and validation cohorts were higher than those of the clinico-pathological and radiomics models. According to the DeLong test, the corresponding *P*-value between the combined radiomics model and the clinical model was less than 0.05 in the validation cohort, and it was also the smallest among several models, suggesting that the combined radiomics nomogram has the strongest significance in predicting thymoma risk stratification compared to other models (Table 4).

**Table 4:** Comparison of the prediction with the combined radiomics nomogram, clinical, and radiomics model

Group	Model 1	Model 2	*P* value
Training	Nomogram	Clinico-radiological	0.026
	Nomogram	Radiomics	0.417
	Clinico-radiological	Radiomics	0.042
Validation	Nomogram	Clinico-radiological	0.018
	Nomogram	Radiomics	0.420
	Clinico-radiological	Radiomics	0.022

**Fig. 5:**
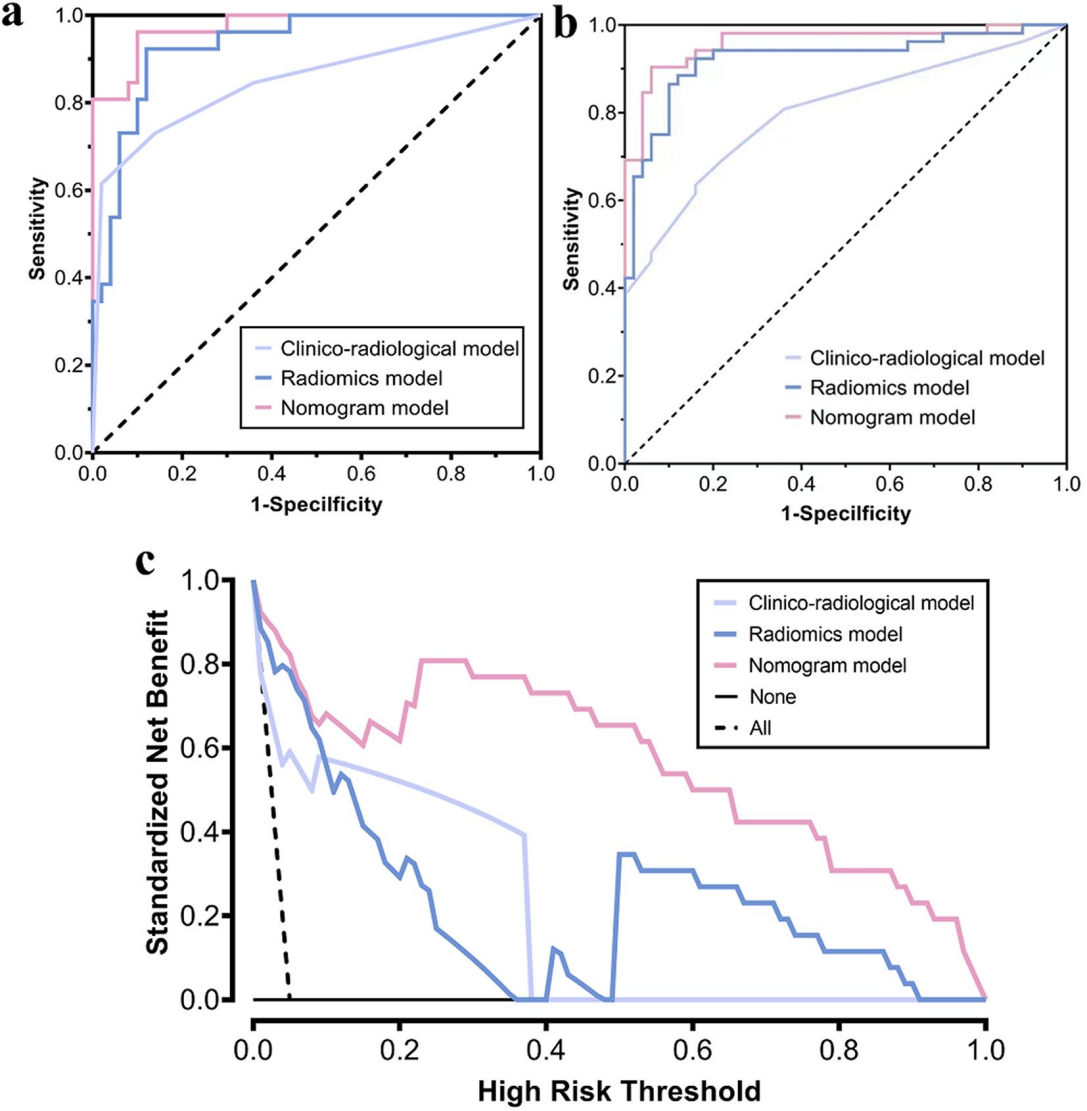
The AUC values for clinico-radiological model, radiomics model and radiomics nomogram (**a**: training cohort, *n* = 125; **b** validation cohort, *n* = 93). Decision curve analysis (DCA) of the clinico-radiological model, radiomics model and radiomics nomogram (**c**)

**Fig. 6:**
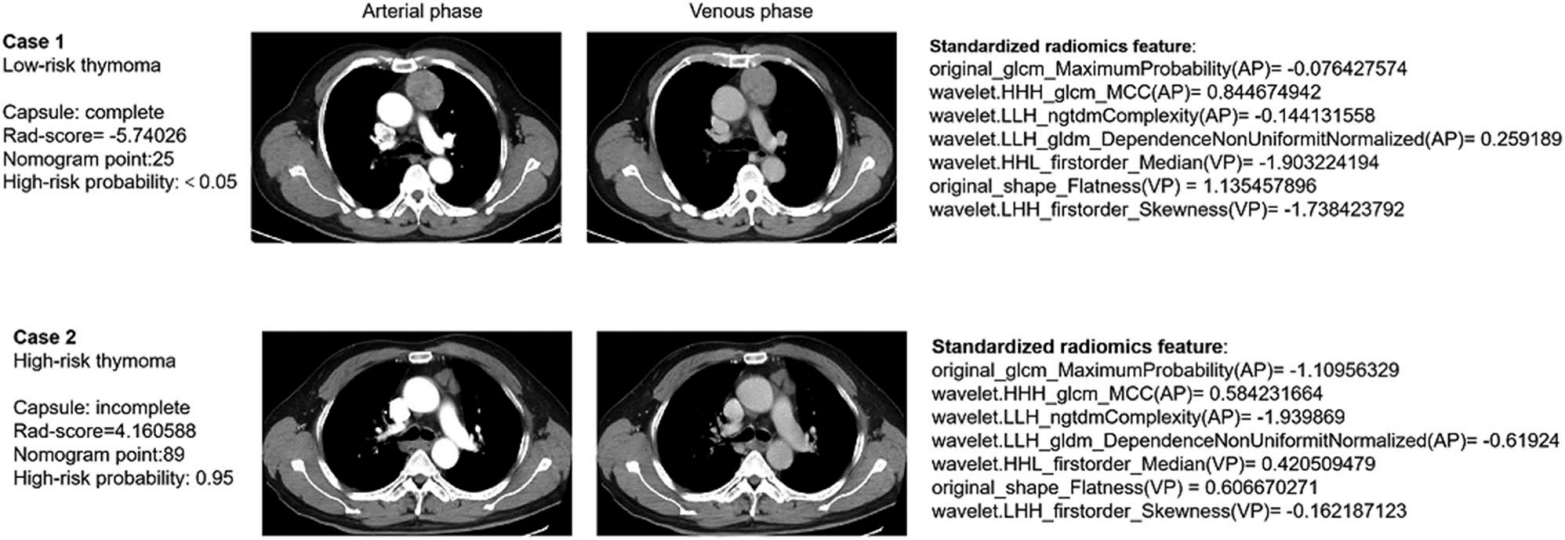
A combined radiomics nomogram was applied in clinical scenarios for pre-operative differentiation between LRT and HRT. Case 1: mediastinal window of axial thin-section enhanced chest CT images in a male with proven diagnosis of low-risk thymoma. Chest CT image shows, in the anterior mediastinum, a round solid mass with complete capsule. The rad-score calculated by nomogram was − 5.74026. The predicted risk value was less than 0.05, which indicated that a low-risk thymoma. Case 2: In a male, CT scan shows an irregular solid nodule with incomplete capsule in the anterior mediastinum. We used the same method to obtain the rad-score (4.160588). The predicted risk value was 0.95, indicating the lesion was a high-risk thymoma

The calibration curves (Fig. 4b, c) demonstrated that the predicted probabilities of the combined radiomics nomogram were closely aligned with the actual clinical observations in both the training and validation cohorts (Hosmer-Lemeshow test, both *P* values > 0.05). The decision curve analysis indicated a higher net benefit for the prediction of thymoma risk stratification than the clinico-radiological model and the radiomics model (Fig. 5). This suggests that the results predicted by our combined radiomics nomogram demonstrated favorable clinical usefulness, and two representative cases were presented to illustrate the application of the radiomics-based nomogram in a clinical scenario (shown in Fig. 6).

## Discussion

With thymomas representing the most common type of tumors in the anterior mediastinum, differentiating their risk categorization is extremely vital due to the rather distinct treatment strategies and clinical prognoses [26]. High-risk thymomas (HRTs) share overlapping CT findings with low-risk thymomas (LRTs), especially when HRTs lack obvious liquefaction and necrosis, making an accurate differential diagnosis challenging using conventional imaging methods [27]. Therefore, how to identify the risk grades of thymomas accurately before surgery is an essential question facing clinicians in planning therapeutic strategies. In this study, we aimed to design an effective program to differentiate HRTs from LRTs. To our best knowledge, no previous study has utilized a radiomics nomogram to predict risk grades for patients with thymomas before surgery. Thus, we developed three prediction models: one clinico-radiological signatures-based model, one radiomics signatures-based model, and one incorporating both. Furthermore, our study successfully demonstrated that the integrated prediction model outperformed the model based on clinico-radiological signatures alone and performed comparably to the radiomics signatures-based model alone in differentiating HRTs from LRTs. The novel radiomics nomogram model could potentially be useful in clinical practice for developing individualized therapeutic schedules.

In the present study, our experimental results show that the CECT-based nomogram prediction model, which integrates clinico-radiological features and radiomics signatures, achieves excellent predictive efficacy for differentiating HRTs from LRTs, with an AUC of 0.960 in patients with thymomas. This was significantly superior to the model based on clinico-radiological features alone (AUC = 0.801) and slightly superior to the model based on the radiomics signature alone (AUC = 0.923), though not reaching statistical significance. Considering that in clinical practice, traditional radiological parameters such as capsule integrity and tumor necrosis severity still play a significant role in differentiating HRTs from LRTs, and the integrated model was more powerful than the former two, we chose the integrated model and represented it with a nomogram.

CT is the first-line imaging technique applied to assess anterior mediastinal masses in clinical routine work. Various strategies have been recommended to differentiate HRTs from LRTs using conventional CT imaging parameters [28]. Ozawa et al. enrolled 31 HRTs and 51 LRTs to determine important CT features for identifying risk categorization. Final results confirmed that HRTs demonstrated more irregular shapes and contours than LRTs. Additionally, HRTs exhibited a higher incidence of invasion into surrounding organs on CT [29]. Han et al. developed and evaluated CT models combining clinical factors and CECT findings for differentiating HRTs from LRTs. They verified that CT imaging features did not significantly differ based on sex or age, but several imaging features demonstrated significant differences between the groups [30]. Sadohara et al. recently stated that combining features based on CT and MRI images, such as contour, capsule, septum, and homogeneous enhancement, were helpful in distinguishing low-risk from high-risk thymomas [31]. In the present study, a clinico-radiological model was built, incorporating demographic information and subjective CE-CT findings. By using incomplete capsule as an independent factor, the clinico-radiological model achieved relatively high AUCs (0.851 in the training cohort; 0.801 in the validation cohort) for differentiating HRTs from LRTs. Hence, consistent with previous findings, our results also indicated that significant clinical factors facilitate accurate diagnosis of thymoma risk categorization.

Presently, radiomics analysis has been widely utilized to comprehensively and non-invasively assess tumor heterogeneity beyond visual evaluation on conventional CT images [32]. Previous research has shown that CT radiomics analysis can differentiate HRTs from LRTs. Yasaka K et al. developed a radiomics model using logistic regression analysis to investigate the feasibility of using CT histogram analysis to differentiate HRTs from LRTs, resulting in an AUC of 0.750 [33]. Compared with the above radiomics investigation on differentiating thymoma risk categorization, our study had several differences and improvements. Firstly, the previous study was carried out on a relatively small sample size, and only histogram signatures were extracted, while radiological features and higher-order features were not applied. Presently, booming radiomics research has provided massive radiomics signatures, allowing for a more integrative assessment of the tumor. In our study, 851 radiomics features were obtained, and finally, 7 features related to tumor homogeneity were chosen as independent factors to develop the radiomics-based model. Numerous selected signatures were high-order filter and wavelet signatures that go beyond conventional texture analysis. Secondly, the area under the ROC analysis for differentiating HRTs from LRTs was just satisfactory (only 0.750) in the previous study, which might have suffered from the small sample size and the relatively low-order features used. Thirdly, we extracted radiomics features from dual-phase images to ensure a more comprehensive evaluation of tumor heterogeneity.

There were still several limitations in this study. Firstly, this study was a retrospective analysis, with selection bias being inevitable. Secondly, all the data were derived from a single center, and multi-center collaboration is imperative for us to collect larger samples to improve diagnostic efficacy in the near future. Thirdly, diagnostic performance may be overestimated in the training cohort due to a lack of external validation.

## Conclusion

In summary, the study developed a CECT-based radiomics nomogram that achieved an optimal differential diagnosis ability between high-risk thymoma (HRT) and low-risk thymoma (LRT) before surgery. As a non-invasive and quantitative method, the radiomics nomogram may serve as an effective tool to assist clinicians in planning personalized treatment strategies.

### Electronic supplementary material

Below is the link to the electronic supplementary material.

Supplementary Material 1

## Data Availability

The datasets generated during and analyzed during the current study are not publicly available due to patient privacy concerns but are available from the corresponding author on reasonable request.

## Abbreviations

CECT: Contrast enhanced CT
LRT: Low-risk thymomas
HRT: High-risk thymomas
WHO: World health organization
DICOM: Digital imaging and communication in medicine
AP: Arterial phase
VP: Venous phase
ROIs: Region of interests
LASSO: Least absolute shrinkage and selection operator
Rad-score: Radiomics score
95% CI: 95% confidence interval
ICC: Intra Class Correlation
VOIs: Volumetric Regions of Interest
ANOVA: One-Way Variance of Analysis
ROC: Receiver Operating Characteristics
AUC: Area Under the Curve

## References

[1] de Jong WK, Blaauwgeers JL, Schaapveld M, et al. Thymic epithelial tumours: a population-based study of the incidence, diagnostic procedures and therapy. Eur J Cancer. 2008;44:123–30. 18068351 10.1016/j.ejca.2007.11.004

[2] Takahashi K, Al-Janabi NJ. Computed tomography and magnetic resonance imaging of mediastinal tumors. J Magn Reson Imaging. 2010;32:1325–39. 21105138 10.1002/jmri.22377

[3] Carter BW, Benveniste MF, Madan R, et al. IASLC/ITMIG staging system and lymph node map for thymic epithelial neoplasms. Radiographics. 2017;37:758–76. 28493800 10.1148/rg.2017160096

[4] Bernard C, Frih H, Pasquet F, et al. Thymoma associated with autoimmune diseases: 85 cases and literature review. Autoimmun rev. 2016;15:82–92. 26408958 10.1016/j.autrev.2015.09.005

[5] Ried M, Marx A, Götz A, et al. State of the art: diagnostic tools and innovative therapies for treatment of advanced thymoma and thymic carcinoma. Eur J Cardiothorac Surg. 2016;49:1545–52. 26670806 10.1093/ejcts/ezv426

[6] Hsu CH, Chan JK, Yin CH et al. Trends in the incidence of thymoma, thymic carcinoma, and thymic neuroendocrine tumor in the United States. PLoS ONE. 2019;14: e0227197. 10.1371/journal.pone.0227197 PMC6938371 31891634

[7] Fukumoto K, Taniguchi T, Ishikawa Y, et al. The utility of [^18^F]-fluorodeoxyglucose positron emission tomography-computed tomography in thymic epithelial tumours. Eur J Cardiothorac Surg. 2012;42:e152–6. 23024234 10.1093/ejcts/ezs527

[8] Kondo K, Yoshizawa K, Tsuyuguchi M, et al. WHO histologic classification is a prognostic indicator in thymoma. Ann Thorac Surg. 201604;77:1183–8. 10.1016/j.athoracsur.2003.07.042 15063231

[9] Qu YJ, Liu GB, Shi HS, et al. Preoperative CT findings of thymoma are correlated with postoperative Masaoka clinical stage. Acad Radiol. 2013;20:66–72. 22981603 10.1016/j.acra.2012.08.002

[10] Rena O, Papalia E, Maggi G, et al. World Health Organization histologic classification: an independent prognostic factor in resected thymomas. Lung Cancer. 2005;50:59–66. 16009453 10.1016/j.lungcan.2005.05.009

[11] Odaka M, Tsukamoto Y, Shibasaki T, et al. Surgical and oncological outcomes of thoracoscopic thymectomy for thymoma. J Vis Surg. 2017;3:54. 29078617 10.21037/jovs.2017.03.18 PMC5637563

[12] Yamazaki M, Oyanagi K, Umezu H, et al. Quantitative 3D shape analysis of CT images of Thymoma: a comparison with histological types. Am J Roentgenol. 2020;214:341–7. 31691609 10.2214/AJR.19.21844

[13] Yang Y, Dong J, Huang Y. Thoracoscopic thymectomy versus open thymectomy for the treatment of thymoma: a meta-analysis. Eur J Surg Oncol (EJSO). 2016;42:1720–8. 27139936 10.1016/j.ejso.2016.03.029

[14] Koçer B, Kaplan T, Günal N, et al. Long-term survival after R0 resection of thymoma. Asian Cardiovasc Thorac Annals. 2018;26:461–6. 10.1177/0218492318778634 29945456

[15] Lee GD, Kim HR, Choi SH, et al. Prognostic stratification of thymic epithelial tumors based on both Masaoka-Koga stage and WHO classification systems. J Thorac Dis. 2016;8:901–10. 27162665 10.21037/jtd.2016.03.53 PMC4842806

[16] Kim BK, Cho BC, Choi HJ, et al. A single institutional experience of surgically resected thymic epithelial tumors over 10 years-clinical outcomes and clinicopathologic features. Oncol Rep. 2008;19:1525–31. 18497960

[17] Jeong YJ, Lee KS, Kim J, et al. Does CT of thymic epithelial tumors enable us to differentiate histologic subtypes and predict prognosis? Am J Roentgenol. 2004;183:283–9. 15269013 10.2214/ajr.183.2.1830283

[18] Ruffini E, Filosso PL, Mossetti C, et al. Thymoma: inter-relationships among World Health Organization histology, Masaoka staging and myasthenia gravis and their independent prognostic significant a single-center experience. Eur J Cardiothorac Surg. 2011;40:146–53. 21093283 10.1016/j.ejcts.2010.09.042

[19] Lambin P, Leijenaar RTH, Deist TM et al. Radiomics: the bridge between medical imaging and personalized medicine. Nat Reviews Clin Oncol 14: 749–62. 10.1038/nrclinonc.2017.141 28975929

[20] Kumar V, Gu Y, Basu S, et al. Radiomics: the process and the challenges. Magn Reson Imaging. 2012;30:1234–48. 22898692 10.1016/j.mri.2012.06.010 PMC3563280

[21] Li B, Xin YK, Xiao G, et al. Predicting pathological subtypes and stages of thymic epithelial tumors using DWI: value of combining ADC and texture parameters. Eur Radiol. 2019;29:5330–40. 30877464 10.1007/s00330-019-06080-4

[22] Ito H, Shimada K, Isogami K, et al. Recurrent thymoma: radiological (CT and FDG-PET) and histological (WHO criteria) features. Radiat Med. 2006;24:292–6. 16958404 10.1007/s11604-005-1541-1

[23] Yang L, Chang J, He X, et al. PET/CT-based radiomics analysis may help to predict neoadjuvant chemotherapy outcomes in breast cancer. Front Oncol. 2022;12:849626. 36419895 10.3389/fonc.2022.849626 PMC9676961

[24] Yang L, Chu W, Li M, et al. Radiomics in Gastric Cancer: First Clinical Investigation to Predict Lymph Vascular Invasion and Survival Outcome using 18F-FDG PET/CT images. Front Oncol. 2022;12:836098. 35433451 10.3389/fonc.2022.836098 PMC9005810

[25] Yang L, Xu P, Zhang Y, et al. A deep learning radiomics model may help to improve the prediction performance of preoperative grading in meningioma. Neuroradiology. 2022;64:1373–82. 35037985 10.1007/s00234-022-02894-0

[26] Zhang GM, Sun H, Shi B, et al. Quantitative CT texture analysis for evaluating histologic grade of urothelial carcinoma. Abdom Radiol. 2017;42:561–8. 10.1007/s00261-016-0897-2 27604896

[27] Moon JW, Lee KS, Shin MH, et al. Thymic epithelial tumors: prognostic determinants among clinical, histopathologic, and computed tomography findings. Ann Thorac Surg. 2015;99:462–70. 25534526 10.1016/j.athoracsur.2014.09.050

[28] Hu YC, Wu L, Yan LF, et al. Predicting subtypes of thymic epithelial tumors using CT: new perspective based on a comprehensive analysis of 216 patients. Sci Rep. 2014;4:6984. 25382196 10.1038/srep06984 PMC4225535

[29] Ozawa Y, Hara M, Shimohira M, et al. Associations between computed tomography features of thymomas and their pathological classification. Acta Radiol. 2016;57:1318–25. 26089525 10.1177/0284185115590288

[30] Han X, Gao W, Chen Y, et al. Relationship between computed tomography imaging features and clinical characteristics, Masaoka-Koga stages, and World Health Organization Histological Classifications of Thymoma. Front Oncol. 2019;9:1041. 31681579 10.3389/fonc.2019.01041 PMC6798238

[31] Sadohara J, Fujimoto K, Müller NL, et al. Thymic epithelial tumors: comparison of CT and MR imaging findings of low-risk thymomas, high-risk thymomas, and thymic carcinomas. Eur J Radiol. 2006;60:70–9. 16766154 10.1016/j.ejrad.2006.05.003

[32] Padda SK, Terrone D, Tian L, et al. Computed tomography features associated with the eighth edition TNM stage classification for thymic epithelial tumors. J Thorac Imaging. 2018;33:176–83. 29219888 10.1097/RTI.0000000000000310 PMC6368176

[33] Yasaka K, Akai H, Nojima M, et al. Quantitative computed tomography texture analysis for estimating histological subtypes of thymic epithelial tumors. Eur J Radiol. 2017;92:84–92. 28624025 10.1016/j.ejrad.2017.04.017

